# The role of CDK4/6 inhibitors in older and younger patients with breast cancer: A systematic review and meta-analysis

**DOI:** 10.1016/j.breast.2023.05.002

**Published:** 2023-05-13

**Authors:** Fausto Petrelli, Lorenzo Dottorini, Giandomenico Di Menna, Karen Borgonovo, Maria Chiara Parati, Carmen Giusy Rea, Mara Ghilardi, Antonio Ghidini, Andrea Luciani

**Affiliations:** a Oncology Unit, ASST Bergamo Ovest, Treviglio, BG, Italy; b IRCCS Istituto Romagnolo per Lo Studio Dei Tumori (IRST) “Dino Amadori”, Meldola, Italy; c Oncology Unit, Casa di Cura Igea, Milano, Italy

**Keywords:** Breast cancer, Elderly, CDK4/6 inhibitors, Metastatic disease, Meta-analysis

## Abstract

**Introduction:**

Cyclin-dependent kinase 4/6 (CDK4/6) inhibitors have an extremely important impact on the treatment of hormone-sensitive breast cancer (BC) and have radically changed the first-line treatment for metastatic disease with increased rates of treatment response, overall survival (OS), and progression-free survival (PFS). We performed a pooled analysis of randomized trials to validate or refute the hypothesis that there is a significant survival benefit of adding anti-CDK4/6 inhibitors to standard endocrine therapy (ET) in older patients with advanced BC.

**Methods:**

We selected only English-language phase II/III randomized controlled trials that compared ET alone with ET with anti-CDK4/6 inhibitors in the treatment of advanced BC, with subgroups reporting the outcomes of elderly patients (usually at least 65 years). The primary endpoint was OS.

**Results:**

The review process led to the inclusion of 12 articles and two meeting abstracts, including a total of 10 trials. The addition of CDK4/6 inhibitors to ET (letrozole or fulvestrant) significantly reduced mortality risk by 20% in younger patients (fixed-effect model; HR 0.80; 95% CI 0.72–0.9; p < 0.01) and 21% in older BC patients (HR 0.79; 95% CI 0.69–0.91; p < 0.01). No OS data were available for patients ≥70 years.

**Conclusion:**

This large, pooled analysis is the first to demonstrate that CDK4/6 inhibitors confer OS and PFS benefits in elderly patients (those aged ≥65 years) with advanced ER + BC and to indicate that it should be discussed with and offered to all patients after geriatric assessment and according to the toxicity profile.

## Highlights

• **Cyclin-dependent kinase 4/6 (CDK4/6) inhibitors have changed the treatment of hormone-sensitive breast cancer (BC)**.
• We performed a pooled analysis to validate the survival benefit of adding anti-CDK4/6 inhibitors in older patients.
• **The review process led to the inclusion of 12 articles and two meeting abstracts, including a total of 10 trials.**
• The addition of CDK4/6 inhibitors significantly reduced the mortality risk by 21% in older BC patients**.**
• This pooled analysis demonstrates that CDK4/6 inhibitors confer OS benefit in elderly patients with advanced ER+ BC.

## 1 Introduction

Breast cancer (BC) is the most common cancer in women, and the majority of new diagnoses are made in older patients, who mainly present with an estrogen receptor (ER)-positive and HER2-negative disease. According to 2022 cancer statistics, more than 80% of invasive BCs cases are diagnosed among women aged 50 years or older, and 91% of deaths occur in this age group. Specifically, half of BC deaths occur in women aged 70 years or older [1].

Cyclin-dependent kinase 4/6 (CDK4/6) inhibitors have an extremely important impact on the treatment of hormone-sensitive disease and have radically changed the first-line treatment for metastatic disease with increased rates of treatment response, overall survival (OS), and progression-free survival (PFS). Three drugs are currently in use for the treatment of ER-positive and HER-2-negative metastatic disease, namely palbociclib, ribociclib, and abemaciclib, all of which have demonstrated a clear advantage in terms of efficacy in the treatment of this pathology.

Older patients are typically underrepresented in clinical trials, and no ad hoc studies are usually conducted for this population; therefore, it is difficult to define parameters such as efficacy, tolerability, and discontinuation specific to this population. Owing to these uncertainties, we performed a pooled analysis of randomized trials to validate or refute the hypothesis that there is a significant survival benefit of adding anti-CDK4/6 inhibitors to standard endocrine therapy (ET) in older patients with advanced BC and to establish its tolerability in this population.

## 2 Material and methods

We performed this systematic review in accordance with the Preferred Reporting Items for Systematic Reviews and Meta-Analyses (PRISMA) guidelines [2]. Using the terms “breast cancer” (palbociclib or ribociclib or abemaciclib) and “elderly” or,” “older” or,” “65 years,” “70 years,” or “75 years,” we searched PubMed, Embase, and the Cochrane Library during February 2023. We selected only English-language phase II–III randomized controlled trials that compared ET alone with ET with anti-CDK4/6 inhibitors in the treatment of advanced BC, with subgroups reporting the outcomes of elderly patients (according to the paper cutoff, usually at least 65 years). In phase I, adjuvant, neoadjuvant, and observational (or real-life) studies were excluded. The primary endpoint was OS. The secondary endpoints were PFS and main toxicities. Quality assessment was performed in duplicate using the Cochrane risk-of-bias (RoB 2) tool for randomized trials. This qualitative assessment evaluates six domains: randomization, allocation concealment, blinding of participants and personnel, blinding of outcome assessment, attrition bias, and selective reporting. Each domain was judged to have a low, unclear, or high risk of bias [3]. Two authors (FP and AG) extracted the following data from the studies: the first author's name; publication year; trial phase; number of enrolled patients; treatment type used in the experimental and control arms; HR for OS and PFS with relative 95% CIs; and the toxicity rates in the subgroup of elderly patients in each treatment arm.

HR for OS and PFS with relative 95% CIs were extracted from each study. Summary HRs were calculated using random- or fixed-effects models depending on the heterogeneity of the included studies. Statistical heterogeneity between the trials included in the meta-analysis was assessed using Cochran's Q test, and inconsistency was quantified using the I^2^ statistic [4]. The homogeneity assumption was considered invalid when the p-values were less than 0.1. When no substantial heterogeneity was observed, the pooled estimate calculated using the fixed-effects model was reported using the inverse-variance method. Where substantial heterogeneity was observed, the pooled estimate based on the random-effects model was reported using the DerSimonian and Laird method that considers both within- and between-study variations [5]. A two-tailed p-value of less than 0.05 was considered statistically significant. The statistical analyses were performed using RevMan software for meta-analysis (v.5.4.1) [6].

## 3 Results

**Fig. 1:**
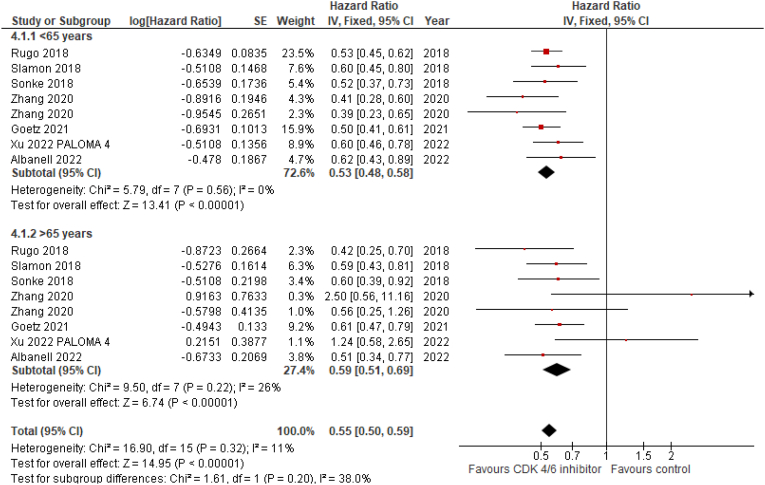
Forest plot for PFS in CDK 4/6 inhibitors vs placebo + endocrine therapy.

**Fig. 2:**
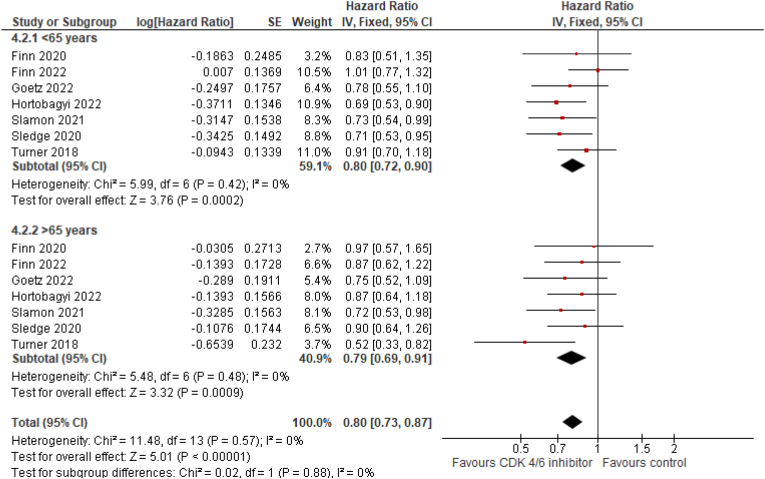
Forest plot for OS in CDK 4/6 inhibitors vs placebo + endocrine therapy.

The review process (Suppl. Fig. 1) led to the inclusion of 12 articles and two meeting abstracts, including a total of 10 trials [[7], [8], [9], [10], [11], [12], [13], [14], [15], [16], [17], [18], [19], [20]]. All studies, except for PALOMA 1 and FLIPPER, were randomized phase III trials that tested the efficacy of palbociclib, ribociclib, and abemaciclib + ET in advanced BC (Suppl. Tab. 1 and suppl. Fig. 1). All studies reported the OS data for 1985 older patients. The addition of CDK 4/6 inhibitors to ET (letrozole or fulvestrant) significantly reduced the mortality risk by 20% in younger patients (fixed-effect model; HR 0.80; 95% CI 0.72–0.9; p < 0.01) and by 21% in older BC patients (HR 0.79; 95% CI 0.69–0.91; p < 0.01) (Fig. 1, Fig. 2). No heterogeneity was observed in the OS analysis (P = 0.57; I^2^ = 0%). Subgroup differences were not statistically significant. The data of OS were only statistically significant in the older population for palbociclib and ribociclib. Similar results were observed for PFS, where these agents reduced the risk of progression by 47% < and >41%, respectively, in patients aged < and >65 years. Data for survival in patients aged ≥70 years were not available for all studies. For PFS analysis, data of PALOMA-2, MONALEESA-2, MONARCH-2 and 3, outcome informations were available for patients aged ≥75 years (pooled HR = 0.53; 95% CI 0.48–0.58) and favored CDK 4/6 + ET arms.

Neutropenia and diarrhea grades (G)3–4 were similar in elderly patients. Only neutropenia G1–4 was significantly higher among the elderly (RR 12.2; 95% CI 9–16.5 vs 24.7; 95% CI 14.1–43.1; p for interaction 0.03). Other G1–4 toxicities (anemia, fatigue, diarrhea, anorexia, vomiting, and nausea) increased similarly in both groups. The treatment discontinuation rates among older patients were 5.5, 9.3, and 16.8% in palbociclib, ribociclib and abemaciclib, respectively (they were 1.5, 7, and 8.7% in patients <65 years).

## 4 Discussion

Patients with advanced ER+/HER2-negative BC who had previously been exposed to ET are candidates for upfront or second-line use of CDK4/6 inhibitors with either an aromatase inhibitor or fulvestrant. Several large phase III trials with long-term follow-up have confirmed their efficacy in terms of PFS and OS in various patient subgroups. The most influential guidelines recommend CDK4/6 inhibitors as the first- or second-line therapy for all postmenopausal women with advanced BC, independent of previous adjuvant therapy, number and site of metastases, performance status, and histology (excluding visceral crises) [21].

A substantial number of enrolled patients were of advanced age (approximately 40–50% of patients enrolled in the PALOMA, MONALEESA, and MONARCH studies). According to the results of our study, it can be concluded that the addition of CDK4/6 inhibitors in older BC patients results in a statistically significant benefit in terms of PFS and reduced mortality. These results are significant because older patients with comorbidities are at greater risk of dying from other causes. In addition, it is not common to observe a significant OS benefit with first-line agents in BC patients due to subsequent lines of therapy during the course of the disease.

Despite a different toxicity profile, neutropenia and diarrhea G3–4 were numerically, but not significantly, higher among the elderly. Only G1–4 neutropenia was significantly worse among older patients than in those aged <65 years. Whether this led to higher discontinuation rates or dose reductions was not described or compared with the control arms in all studies. All other grade toxicities, such as nausea, vomiting, fatigue, anorexia, anemia, and diarrhea, were similar between the two subgroups. In the PALOMA studies, few patients discontinued treatment because of adverse events with respect to other inhibitors. Clinically relevant diarrhea (G2–3) occurred more frequently in the two groups with older patients (<65 years, 39.5%; 65–74 years, 45.2%; ≥75 years, 55.4%), and discontinuation due to adverse events was higher in the 65–74 and ≥75 subgroups than in the <65 group. Whether this led to the insignificant gain in OS observed in the abemaciclib studies is not known. Overall, only ribociclib and palbociclib maintained a survival benefit, even in elderly patients.

Various regimens are available for HER-2-negative advanced BC, and CDK4/6 inhibitors performed best in terms of PFS, according to a network meta-analysis published by Giuliano et al., in 2019 [22]. OS was not available for the majority of the studies at the time of publication, so the primary endpoint was not mortality. We confirmed the efficacy of these agents in terms of both PFS and OS, even in an older population with a similar toxicity profile.

Our findings reflect those of a well-selected patient profile enrolled in clinical trials. Furthermore, the age limit for older patients was 65 years for all of the included studies. Other authors have used a higher cutoff for the elderly population in a retrospective case series. In an analysis of 605 patients treated with palbociclib in the United States (US), 92 were aged ≥70 years old [23]. They had a significantly increased number of dose reductions and dose delays compared with younger patients, but they had a significantly improved PFS compared with the younger cohort. Although the follow-up period was not mature, age did not affect OS. In another real-world series, Wilkie et al. found that dose reductions among women older and younger than 70 years were not significantly different [24]. Olazagasti et al. found similar PFS and OS between age groups in a geriatric series of 202 patients older than 70 years without significant differences in neutropenia or thrombocytopenia toxicity [25]. In a retrospective series from Singapore, elderly patients had shorter PFS and were more likely to require dose modifications [26]. Finally, in an FDA pooled analysis of three randomized trials (PALOMA 2, MONALEESA 2, and MONARCH 3) in patients aged 70 years and older (n = 456), the HR for PFS of the combination of CDK 4/6 inhibitors + letrozole compared with letrozole alone was 0.52 (95% CI, 0.38 to 0.70), similar to result attained in our analysis with patients older than 65 years [27]. Although G3/4 toxicities were higher (84%) in patients aged 70 years and older than in those younger than 70 years (72%), older patients reported a decline in quality-of-life (QoL) measures in both arms. Unfortunately, data on OS were not available at time of FDA analysis. Currently, the American Society of Clinical Oncology guidelines recommend geriatric evaluation for cancer patients older than 65 years, and we consider it appropriate to use this cutoff for evaluating the efficacy of CDK 4/6 in elderly patients [28]. CGA predicts greater susceptibility to QoL impairment in older adults with cancer. Correlation data of age and QoL in elderly patients treated with CDK 4/6 inhibitors are lacking in major phase III studies [29,30], and SIOG guidelines suggest that vulnerabilities discovered with CGA may affect the decision to use CDK4/6 inhibitors in frail adults. In certain cases, ET alone without CDK4/6 inhibitors may be used to minimize the impact of potential toxic interactions on QoL [31]. In a review of Di Lauro et al. [31], they found that CDK4/6 inhibitors did not significantly affect QoL despite methodological heterogeneity. There was also a positive trend toward pain improvement with CDK4/6 inhibitors plus ET. A valuable index for estimating the usefulness of each drug is the likelihood of being helped or harmed by these drugs. When >1, the drug is considered more useful than harmful. Although this index was not evaluated only in the elderly, it may guide treatment decision-making. Mastrantoni et al. identified ribociclib as better for diarrhea, fatigue, dose reduction, and discontinuations, whereas abemaciclib was better for neutropenia, febrile neutropenia, and transaminase increase [32]. Palbociclib is preferred to reduce the risk of dose interruption or reduction, as shown in elderly or frail patients. When the survival advantage has to be weighed based on tolerability, palbociclib can be considered a reasonable choice.

The results also reflect those observed in large real-world studies in which more unselected patients were included (e.g., older patients or those with poorer performance status) [[33], [34], [35], [36], [37]]. A retrospective study by De Michele et al. showed that among 1430 patients treated with first-line palbociclib in combination with letrozole or letrozole alone, the effect on OS was even greater than the value attained in our meta-analysis (HR = 0.55, 95% CI 0.4–0.77) in patients older than 70 years. In the large phase IIIb study, CompLEEment, one-third of the enrolled patients were aged >65 years. However, no subgroup analysis has been conducted on this population. In the IRIS database, almost 3000 patients were treated with palbociclib in combination with aromatase inhibitors/fulvestrant, and 48% were older than 65 years. Overall, a 1-year PFS and OS were similar in younger and older patients, and the author explained that this was because some patients started treatment at a lower-than-recommended dose because of their age. In these real-world studies, the rate of adverse events appears to be lower than that in randomized studies, but proactive management with upfront dose mitigation may explain the higher rates of dose reduction. Recently, a large observational real‐world study demonstrated that ET in combination with CDK4/6 inhibitor therapy significantly improved OS in an older population of patients with ER+/HER2– de novo advanced BC, who were enrolled in the US Medicare system. Despite the retrospective nature of the data, this study corroborated our findings.

Our pooled analysis has several limitations. First, publication bias may have affected the subgroup analysis because age was not used as a stratification factor. Furthermore, geriatric comorbidities and geriatric evaluations were not documented or extensively assessed in any of the studies. Third, we did not have individual patient data; therefore, we could not calculate the outcomes according to stage or risk class. Fourth, patients enrolled in clinical trials were generally healthier than the real-world population, which may have influenced the results of this meta-analysis by excluding frail and vulnerable patients encountered in clinical practice. Finally, the pooled analysis included patients treated for de novo or recurrent disease or disease progression after the first-line/adjuvant aromatase inhibitors. Nevertheless, our meta-analysis provides the first pooled estimates of CDK4/6 inhibitor efficacy in elderly patients with advanced BC, and the final results were consistent in younger and older subjects in both PFS and OS analyses.

In conclusion, this large, pooled analysis is the first to demonstrate that CDK4/6 inhibitors confer OS and PFS benefits in elderly patients (those aged ≥65 years) with advanced ER + BC and to indicate that it should be discussed with and offered to all patients after geriatric assessment and according to the toxicity profile. Owing to the lack of individual patient data, it is not possible to make clear recommendations about possible subgroups where these agents are not effective. Appropriate dose reduction/interruption may mitigate more common adverse events without compromising the efficacy. Thus, almost all elderly patients are candidates for these agents. Therefore, dedicated clinical trials in geriatric populations are required.
