# Supplementary material for: The role of CDK4/6 inhibitors in older and younger patients with breast cancer: A systematic review and meta-analysis
Source: Breast. 2023 May 13;71:138–42. doi: 10.1016/j.breast.2023.05.002 (PMC10512091; doi:10.1016/j.breast.2023.05.002)
Supplement: Multimedia component 1 [file mmc1.docx]

**Identification of studies via databases and registers**

Records removed *before screening*:

Duplicate records removed (n =147)

Records marked as ineligible (n = 535)

Records removed for non English language (n = 55)

Records identified from:

Pubmed (n = 40)

Other databases (n = 858)

**Identification**

Records excluded because were phase II studies (n = 89)

Records screened

(n = 161)

Reports excluded because they were studies in early stages of disease or HER2 + breast cancer (n =37)

Reports sought for retrieval

(n = 72)

**Screening**

Reports excluded:

Included older version of included trials

(n = 25)

Reports assessed for eligibility

(n = 35)

Studies included in review

(n = 10)

Reports included in quantitative sinthesis (n = 10)

**Included**

**Suppl. Fig. 1 flow diagram of included studies**
