# Supplementary material for: The role of CDK4/6 inhibitors in older and younger patients with breast cancer: A systematic review and meta-analysis
Source: Breast. 2023 May 13;71:138–42. doi: 10.1016/j.breast.2023.05.002 (PMC10512091; doi:10.1016/j.breast.2023.05.002)
Supplement: Multimedia component 2 [file mmc2.docx]

| Author/year | Type and line of study | Median follow up (months) | Standard arm | Experimental arm | Cut off age/n° elderly pts | All G1-4 toxicities % exp vs ctr arm  (>65 years) | All G3-4 toxicities % exp vs ctr arm  (>65 years) | G3-4 neutropenia exp vs ctr arm %  (>65 years) | G3-4 diarrhea exp vs ctr arm %  (>65 years) | HR PFS (95%CI) | HR OS (95%CI) | Bias |
| --- | --- | --- | --- | --- | --- | --- | --- | --- | --- | --- | --- | --- |
| Sonke/2018  Hortobagyi/2022  (MONALEESA-2) | Phase 3 | 79.2 | Letrozole + placebo | Letrozole + ribociclib | 65/295 | 99 vs 97 | 87 vs 39 | 60 vs 0 | 2 vs 1 | 0.60  (0.39-0.92) | 0.87  (0.64-1.18) | Low |
| Slamon/2018  Slamon/2021  (MONALEESA-3) | Phase 3 | 56.3 | Fulvestrant + placebo | Fulvestrant + ribociclib | 65/339 | - | - | - | - | 0.59  (0.43-0.81) | 0.72  (0.53-0.98) | Low |
| Sledge/2020  Goetz/2021  (MONARCH-2) | Phase 3 | 47.7 | Fulvestrant + placebo | Fulvestrant + abemaciclib | 65/245 | - | - | 24.8 | 14.5 | - | 0.90  (0.64-1.26) | Low |
| Goetz/2021  Goetz/2022  (MONARCH-3) | Random | 26.7 | NSAI + placebo | NSAI + abemaciclib | 65/222 |  |  |  |  | 0.61  (0.47-0.69) | 0.75  (0.52-1.09) | Low |
| Zhang/2020  (MONARCH PLUS) | Phase 3 | 16 | NSAI + placebo  Fulvestrant + placebo | NSAI + abemaciclib  Fulvestrant + abemaciclib | 65/106 | - | - | - | - | 2.5  (0.56-11.1)  0.56  (0.24-1.26) | - | Low |
| Rugo/2018  Finn/2020  (PALOMA-1) | Phase 2 random | 64.7 | Letrozole | Letrozole + palbociclib | 65/92 | 99.5 vs 91.6 | 80.5 vs 22.1 | 68.4 vs 0.4 | 1.6 vs 0.4 | 0.42  (0.25-0.70) | 0.97  (0.57-1.65) | Uncertain |
| Rugo/2018  Finn/2022  (PALOMA-2) | Phase 3 | 90 | Letrozole + placebo | Letrozole + palbociclib | 65/262 |  |  |  |  | - | 0.87  (0.62-1.22) | Low |
| Rugo/2018  Turner/2018  (PALOMA-3) | Phase 3 | 44.8 | Fulvestrant + placebo | Fulvestrant + palbociclib | 65/129 |  |  |  |  | - | 0.52  (0.33-0.82) | Low |
| Xu/2022  (PALOMA-4) | Phase 3 | 52.8 | Letrozole + placebo | Letrozole + palbociclib | 65/38 | - | - | - | - | 1.24  (0.58-2.65) | - | Low |
| Albanell/2021  (FLIPPER) | Phase 2 random | 28.6 | Fulvestrant + placebo | Fulvestrant + palbociclib | 65/92 | - | - | - | - | 0.51  (0.34-0.75) | - | Uncertain |

HR, hazard ratio; PFS, progression-free survival; OS, overall survival; NSAI, non-steroidal aromatase inhibitor; UTI, urinary tract infection; ALT, alanine aminotransferase; AST, aspartate aminotransferase; VTE, venous thromboembolic event; ILD, interstitial lung disease

**Tab.1 Characteristics of the studies included in the analysis**
